# Supplementary material for: Strong interactions between solitons and background light in Brillouin-Kerr microcombs
Source: Nat Commun. 2024 Feb 23;15:1661. doi: 10.1038/s41467-024-46026-z (PMC10891115; doi:10.1038/s41467-024-46026-z)
Supplement: Supplementary file 1 — Supplementary Information [file 41467_2024_46026_MOESM1_ESM.pdf]

Supplementary Information for

Strong interactions between solitons and background light in Brillouin-Kerr microcombs

Menghua Zhang[1,2], Shulin Ding[1,2], Xinxin Li[1,2], Keren Pu[1], Shujian Lei[1], Min Xiao[1], and Xiaoshun Jiang[1]*

[1]National Laboratory of Solid State Microstructures, College of Engineering and Applied Sciences, School of Physics, Collaborative Innovation Center of Advanced Microstructures, Nanjing University, Nanjing 210093, China.

[2]These authors contributed equally: Menghua Zhang, Shulin Ding, Xinxin Li.

*To whom correspondence should be addressed: jxs@nju.edu.cn.

I. Interaction between the continuous-wave background and the Kerr microcombs

Basing upon our previous work[1], we built up the following theoretical model to describe the generation of the Brillouin-Kerr frequency comb demonstrated in the experiments. That is, we utilized nonlinear coupled-mode equations to delineate the involving processes of the Kerr nonlinearity and the stimulated Brillouin scattering (SBS) as well as the interaction between them. By applying the rotating-wave approximation, the evolution of the Brillouin-Kerr frequency combs thus obeys the following set of the coupled-mode equations,

$$\frac{da_+}{dt} = \left(-i\Delta\omega_p - \frac{\gamma_+}{2}\right) a_+ - ig_b a_{-,0} b + \sqrt{\kappa_+} s_{in}, \tag{S1}$$

$$\frac{da_{-,n}}{dt} = \left(-i\Delta\omega_{b,n} - \frac{\gamma_-}{2}\right) a_{-,n} - i\delta_{0,n} g_b a_+ b^* - ig_K \sum_{k,l,m} \delta_{0,n-(k-l+m)}\, a_{-,k} a_{-,l}^* a_{-,m}, \tag{S2}$$

$$\frac{db}{dt} = \left(-i\Delta\Omega_m - \frac{\gamma_m}{2}\right) b - ig_b a_+ a_{-,0}^*, \tag{S3}$$

with $n, k, l, m \in \{0, \pm1, \pm2, \ldots\}$. Equations (S1) - (S3) follow the similar definitions used in our previous work[1]. Specifically, t stands for the slowly varying time variable compared to the cavity roundtrip, $\delta_{0,n}$ is the Kronecker delta, n is the index of comb modes in the Brillouin mode family, a_+, $a_{-,0}$, $a_{-,n}$, and b represent the amplitudes of the pump, Brillouin, comb, and acoustic waves, respectively. In Eqs. (S1)-(S3), $\Delta\omega_p = \omega_p - \omega_+$, $\Delta\omega_{b,0} = \omega_b - \omega_{-,0}$, $\Delta\omega_{b,n} = \omega_{b,n} - \omega_{-,n}$, and $\Delta\Omega_m = \Omega_m - \Omega_0$ are, respectively, the frequency detuning of the pump, Brillouin, n-th comb, and acoustic modes defined by their corresponding resonant frequencies ω_+, $\omega_{-,0}$, $\omega_{-,n}$, and Ω_0, where ω_p, $\omega_{b,0}$, $\omega_{b,n}$, and Ω_m denote, respectively, the angular frequencies of input pump, Brillouin, comb and acoustic waves; γ_+, γ_-, and γ_m are the decay rates of the pump, Brillouin (comb), acoustic modes, respectively. Also, the resonance frequencies of the comb modes can be written as: $\omega_{-,n} = \omega_{-,0} + D_1 n + \frac{1}{2} D_2 n^2$, with a Taylor expansion around the resonant frequency of the Brillouin mode $\omega_{-,0}$, where

D_1 and D_2 stand for the free spectral range (FSR) and the second-order dispersion, respectively. In addition, we can also calculate the amplitude of transmitted pump, Brillouin, as well as the generated comb waves from the input-output relation,

$$s_{out,+} = s_{in} - \sqrt{\kappa_+} a_+, \quad \text{(S4)}$$

$$s_{out,-} = \sqrt{\kappa_-} \sum_n a_{-,n}\, e^{i(nD_1 + \Delta\omega_{b,n})\tau}, \quad \text{(S5)}$$

where τ is the fast time scale and κ_- stands for the external coupling rate of Brillouin mode or any comb modes.

To reveal the interaction between the continuous-wave (CW) background light and the Kerr-comb formation in generating the Brillouin-Kerr microcomb, we notice that the CW background light in fact comes from the intracavity Brillouin laser. To this end, we first look at the effect of the Kerr comb formation on the dynamics of the Brillouin wave using Eq. (S2), which is,

$$\frac{da_{-,0}}{dt} = \left(-i\Delta\omega_{b,0} - \frac{\gamma_-}{2}\right) a_{-,0} + F_{driv} + F_{comb}, \quad \text{(S6)}$$

where $F_{driv} = -ig_b a_+ b^*$ is the driving term for the Brillouin laser generation and $F_{comb} = -ig_K \sum_{k,l,m} \delta_{0,k-l+m}\, a_{-,k} a^*_{-,l} a_{-,m}$ accounts the contribution from the Kerr nonlinearity. To show how the comb state formation affects the Brillouin lasing, we can rewrite F_{comb} as,

$$F_{comb} = (-i\Delta\omega_{comb} - \frac{\gamma_{comb}}{2}) a_{-,0}, \quad \text{(S7)}$$

in terms of the extra frequency detuning $\Delta\omega_{comb}$ and the extra decay rate γ_{sol} induced by Kerr-comb formation. Here, we find,

$$\Delta\omega_{comb} = g_K \left(\left|a_{-,0}\right|^2 + 2\sum_{k\neq 0}\left|a_{-,k}\right|^2\right) - iIm\left(F_{comb,para}/a_{-,0}\right), \quad \text{(S8)}$$

$$\frac{\gamma_{comb}}{2} = -Re\left(F_{comb,para}/a_{-,0}\right), \quad \text{(S9)}$$

where, $F_{comb,para} = -ig_K \sum_{k,m\neq 0} \delta_{0,k-l+m}\, a_{-,k} a^*_{-,l} a_{-,m}$ adds up all optical-parametric-process terms of F_{comb}, $Re(\ldots)$ and $Im(\ldots)$ stand for the real and imaginary parts of the number, respectively. From Eqs. (S8) and (S9), one can see that all terms of F_{sol} will contribute to the extra detuning $\Delta\omega_{comb}$. However, only the parametric process will lead to the extra decay rate γ_{comb} of the Brillouin mode, which physically means the energy of intracavity Brillouin laser will leak into other comb modes via optical parametric process.

By assuming the steady-state approximation to Eq. (S3), i.e., setting $\frac{db}{dt} = 0$, we obtain,

$$b = -\frac{ig_b a_+ a^*_{-,0}}{i\Delta\Omega_m + \frac{\gamma_m}{2}}. \quad \text{(S10)}$$

Now, substituting Eqs. (S7) and (S10) into Eq. (S6) yields,

$$\frac{da_{-,0}}{dt} = \left(-i\Delta\omega_{b,0} - i\Delta\omega_{comb} - \frac{\gamma_-}{2} - \frac{\gamma_{comb}}{2}\right) a_{-,0} + (i\Delta\Omega_m + \frac{\gamma_m}{2}) \frac{g_b^2}{\Delta\Omega_m^2 + \frac{\gamma_m^2}{4}} |a_+|^2 a_{-,0} \quad .$$

(S11)

Similarly, by setting $\frac{da_{-,0}}{dt}=0$ in Eq. (S11), the resulting equation allows us to rewrite the detuning of the Brillouin wave,

$$\Delta\omega_{b,0}=\Delta\omega'_{b,0}+\Delta\omega''_{b,0}, \tag{S12}$$

where $\Delta\omega'_{b,0}=-\frac{\gamma_m}{\gamma_m+\gamma_-}\Delta\omega_{comb}$ and $\Delta\omega''_{b,0}=\frac{\gamma_-}{\gamma_m+\gamma_-}\left(\Delta\omega_0+\Delta\omega_p\right)$ with $\Delta\omega_0=\omega_+-\omega_{-,0}-\Omega_0$ being the resonant frequency mismatch among the three modes. Since in a microcavity Brillouin laser $\gamma_m\gg\gamma_-$, this immediately leads to $\Delta\omega'_{b,0}\approx-\Delta\omega_{comb}$. Physically, this result suggests that the Kerr-comb formation will shift the frequency of the Brillouin laser with the magnitude approximately equal to the extra frequency detuning induced by the Kerr-comb formation. Therefore, the total frequency detuning term in Eq. (S6) will satisfy $\Delta\omega_{b,0}+\Delta\omega_{comb}\approx\Delta\omega''_{b,0}$, which indicates that the extra frequency detuning $\Delta\omega_{comb}$ affects little on the Brillouin laser.

In order to have a further understanding of how the extra decay rate γ_{comb} induced by Kerr-comb generation affects the intracavity energy of the circulating pump and Brillouin waves, we can derive the following expression for the intracavity energy of the pump wave by balancing the gain and loss terms in Eq. (S11). That is,

$$|a_+|^2=(\gamma_-+\gamma_{comb})\frac{\Delta\Omega_m^2+\frac{\gamma_m^2}{4}}{g_b^2\gamma_m}. \tag{S13}$$

By recalling the phase-matching condition in the SBS process, we have,

$$\Delta\omega_b+\Delta\omega_{comb}+\Delta\Omega_m=\Delta\omega_0+\Delta\omega_p. \tag{S14}$$

Since $\gamma_m\gg\gamma_-+\gamma_{comb}$ is generally satisfied during the microcavity Brillouin lasing, this gives rise to $\Delta\Omega_m\gg\Delta\omega_b+\Delta\omega_{comb}$ and consequently allows us to approximate Eq. (S14) as

$$\Delta\Omega_m\approx\Delta\omega_0+\Delta\omega_p. \tag{S15}$$

By plugging this result into Eq. (S13), one can readily reach the following equation,

$$|a_+|^2\approx(\gamma_-+\gamma_{sol})\frac{(\Delta\omega_0+\Delta\omega_p)^2+\frac{\gamma_m^2}{4}}{g_b^2\gamma_m}. \tag{S16}$$

In the steady state (setting $\frac{da_+}{dt}=0$ and $\frac{da_{-,0}}{dt}=0$), utilizing Eqs. (S1), (S4), and (S7) and eliminating b, we can acquire[2],

$$|s_{in}|^2-\left|s_{out,+}\right|^2=\gamma_{+i}|a_+|^2+(\gamma_-+\gamma_{comb})\left|a_{-,0}\right|^2, \tag{S17}$$

with $\gamma_{+i}=\gamma_+-\kappa_+$ being the intrinsic decay rate of the pump mode inside the cavity. By presume s_{in} to be a real number for the sake of simplicity, from Eq. (S17), one can have,

$$\left|a_{-,0}\right|^2=\frac{2\sqrt{\kappa_+}s_{in}}{\gamma_-+\gamma_{comb}}\cos[\mathrm{Arg}(a_+)]|a_+|-\frac{\gamma_+}{\gamma_-+\gamma_{comb}}|a_+|^2, \tag{S18}$$

where we have used $\mathrm{Arg}(a_+)$ to represent the principle value of the argument a_+. With the help of Eq. (S16), we can now have a quantitative estimation on the intracavity energy of the Brillouin wave, which is,

$$\left|a_{-,0}\right|^2 \approx \frac{2\sqrt{\kappa_+}s_{in}}{\sqrt{\gamma_- + \gamma_{comb}}}\cos[\mathrm{Arg}(a_+)]\sqrt{\frac{[(\Delta\omega_0+\Delta\omega_p)^2+\frac{\gamma_m^2}{4}]}{g_b^2\gamma_m} - \frac{\gamma_+[(\Delta\omega_0+\Delta\omega_p)^2+\frac{\gamma_m^2}{4}]}{g_b^2\gamma_m}}. \quad \text{(S19)}$$

In the steady-state approximation of the Brillouin lasing, the term $\cos[\mathrm{Arg}(a_+)]$ changes negligibly. With this in mind, from Eqs. (S16) and (S19), one can immediately deduce that the intracavity energy of the pump wave will increase but the energy of any Brillouin wave will decrease, owing to the enlarged nonlinear decay rate γ_{comb} during the formation of a Kerr comb.

According to the theoretical framework of dissipative Kerr-soliton formation in microcavity[3,4], the solution for the CW background light, as a fixed quantity, originates from the detuning term $\Delta\omega_p'$ and driving term defined by the external pump laser, $F_{driv}' = \sqrt{\kappa_+'}s_{in}'$, where κ_+' is the external coupling rate of the input pump field with the amplitude s_{in}'. Thereby, if the pump power, frequency detuning and the coupling condition of the cavity modes are all fixed, $\Delta\omega_p'$ and F_{driv}' will remain unchanged. Different from this conventional picture, in our Brillouin-Kerr soliton generation the detuning term $\Delta\omega_{b,0}$ and driving term F_{driv} in Eq. (S6) is variable instead fixed. With the use of Eqs. (S10) and (S13), F_{driv} can be expressed as,

$$F_{driv} = \sqrt{\Delta\Omega_m^2 + \frac{\gamma_m^2}{4}\frac{\gamma_{comb}+\gamma_-}{\gamma_m}}\,a_{-,0}. \quad \text{(S20)}$$

Physically, Eq. (S20) states that the variation of the driving term F_{driv} stems from the amplitude change of the Brillouin wave $a_{-,0}$ as well as the extra decay rate γ_{comb} induced by the generation of Kerr combs. From the theoretical works[3,4], we notice that the amplitude of the CW background field A_{back} is the solution (with the lowermost solution) of the following equation,

$$A_{back} = \frac{F_{driv}}{(\Delta\omega_{b,0}+g_K|A_{back}|^2)i+\frac{\gamma_-}{2}}. \quad \text{(S21)}$$

Now, basing upon Eqs. (S20) and (S21), we can deduce that when $a_{-,0} \to 0$, $F_{driv} \to 0$ and then $A_{back} \to 0$. Due to this chain effect, if γ_{comb} induced by the Kerr-comb generation becomes very large (in comparison to the decay rate of the Brillouin mode γ_-), in accordance with Eq. (S19) the intracavity energy of the Brillouin wave will be greatly reduced and this reduction will further damp the amplitude of the CW background field in a remarkable way.

After understanding how the energy exchange between the background light and soliton formation, we are ready to proceed the discussions on the monostable single-soliton generation. In our previous work[1], we have demonstrated that the Kerr solitons can be directly generated with the intracavity Brillouin laser just above the optical parametric oscillation (OPO) threshold of the Brillouin mode. In this case, any reductions of intracavity energy of the background field will lead the field to below the threshold. This will direct result in a strong interaction between the generated Kerr comb and CW background field, which stabilizes the generated comb into a soliton state. Under this assumption, we have,

$$\left|a_{-,0}\right|_{\gamma_{comb}=0}\Big|^2 \approx \frac{2\sqrt{\kappa_+}s_{in}}{\sqrt{\gamma_-}}\cos\left[\mathrm{Arg}\left(a_+|_{\gamma_{comb}=0}\right)\right]\sqrt{\frac{[(\Delta\omega_0+\Delta\omega_p)^2+\frac{\gamma_m^2}{4}]}{g_b^2\gamma_m}} - \frac{\gamma_+\left[(\Delta\omega_0+\Delta\omega_p)^2+\frac{\gamma_m^2}{4}\right]}{g_b^2\gamma_m} \approx \frac{\gamma_-}{2g_K}. \tag{S22}$$

Here, $a_{-,0}\big|_{\gamma_{comb}=0}$ $(a_+|_{\gamma_{comb}=0})$ denotes the amplitude of Brillouin (pump) wave before the Kerr-soliton formation. From Eq. (S20), it is straightforward to find

$$2\sqrt{\kappa_+}s_{in}\cos\left[\mathrm{Arg}\left(a_+|_{\gamma_{comb}=0}\right)\right]\sqrt{\frac{\left[(\Delta\omega_0+\Delta\omega_p)^2+\frac{\gamma_m^2}{4}\right]}{g_b^2\gamma_m}} = \frac{\sqrt{\gamma_-}\gamma_-}{2g_K} + \frac{\sqrt{\gamma_-}\gamma_+\left[(\Delta\omega_0+\Delta\omega_p)^2+\frac{\gamma_m^2}{4}\right]}{g_b^2\gamma_m}, \tag{S23}$$

To ease the discussions, we assume $\cos\left[\mathrm{Arg}\left(a_+|_{\gamma_{comb}=0}\right)\right] \approx \cos[\mathrm{Arg}(a_+)]$ and insert Eq. (S23) into Eq. (S19). In this way, we obtain the following intracavity energy of the Brillouin wave,

$$\left|a_{-,0}\right|^2 \approx \frac{\sqrt{\gamma_-}}{\sqrt{\gamma_-+\gamma_{comb}}}\frac{\gamma_-}{2g_K} + \frac{\sqrt{\gamma_-}}{\sqrt{\gamma_-+\gamma_{comb}}}\frac{\gamma_+[(\Delta\omega_0+\Delta\omega_p)^2+\frac{\gamma_m^2}{4}]}{g_b^2\gamma_m} - \frac{\gamma_+[(\Delta\omega_0+\Delta\omega_p)^2+\frac{\gamma_m^2}{4}]}{g_b^2\gamma_m}, \tag{S24}$$

Equation (S24) is one of the key results that we have achieved in this reported work and it also sets up the basis for realizing the monostable single-soliton state generation. In other words, this result enables a unique mechanism to reliably form monostable single soliton. To see how this works, from Eq. (S24) one can see that the intracavity Brillouin energy $\left|a_{-,0}\right|^2$ is inversely determined by the only variable γ_{comb}, and any increase of γ_{comb} will alternatively decrease this energy. This monotonic reduction effect becomes more obvious if γ_- is comparable to or smaller than γ_{comb}. This in turn implies that, in an ultra-high-Q cavity, the generation of Kerr solitons will remarkably reduce the intracavity energy of the Brillouin wave. Moreover, if the value of γ_{comb} is sufficiently large, $\left|a_{-,0}\right|^2$ will become near zero, which accordingly provides a physical means to switch off the Brillouin laser and hence terminates the Kerr soliton formation. Meanwhile, the parametric gain obtained by the solitons as they propagate over the CW background field will be dramatically lessened, and will become too weak to support the soliton propagation. In short, during this process, the increase of the soliton number decreases the intracavity energy of the Brillouin field monotonically but enlarges the intracavity energy of the solitons along with increasing the decay rate of the Brillouin laser. Therefore, in line with the above analysis, if we could properly preset the intracavity energy of the Brillouin wave before forming the Kerr solitons to be small, the system energy can be adjusted to only support the generation of the single-soliton state and its propagation. Alternatively, creation of any state beyond a single soliton will substantially augment the decay rate experienced by the Brillouin wave, hence dampening the CW background field amplitude significantly. As a result, the parametric gain supplied by the CW background field will become too weak to enable any multi-soliton excitation as well as their propagation.

II. Numerical simulations on formation of monostable single-soliton state

After theoretically discussing the strong interaction between the background light and the soliton generation and the resulting mechanism of monostable single-soliton formation in the preceding section, here we would like to confirm these results through numerical simulations so as to reach a better understanding of the observed single-soliton Brillouin-Kerr combs in this work. To this end, in Fig. 3a, b of the main text we first present our numerical calculations for the Brillouin-Kerr single-soliton formation using the experimental parameters. We perform the numerical simulations in the light of Eqs. (S1) - (S3) by applying the fourth-order Runge-Kutta method[1]. In the simulations, we choose 512 modes as the comb modes and introduce a computation-efficient algorithm to fast evaluate the cubic nonlinear terms, $g_K \sum_{k,l,m} \delta_{0,n-(k-l+m)}\, a_{-,k} a^*_{-,l} a_{-,m}$ [5]. Additionally, we set the average intracavity energies of the Brillouin wave and all comb waves to be zero at the initial moment, and add weak noise onto each mode to initiate the Brillouin lasing and the Kerr comb generation in the simulations. Specifically, in our numeric we adopt the following measured parameters: the second-order dispersion parameter $D_2 = 2\pi \times 23.7$ kHz, the intrinsic optical decay rates $\gamma_{i+} = 2\pi \times 1.32$ MHz and $\gamma_{i-} = 2\pi \times 0.36$ MHz, and the external optical coupling rates $\kappa_+ = 2\pi \times 0.33$ MHz and $\kappa_- = 2\pi \times 0.09$ MHz along with the input pump power $|s_{in}|^2 = 18.0$ mW. Moreover, we set the pump frequency detuning $\Delta\omega_p = 2\pi \times 0.72$ MHz, the decay rate of the acoustic mode $\gamma_m = 2\pi \times 30$ MHz, and the resonance frequency mismatch among three involved modes to be $\Delta\omega_0 = -2\pi \times 20.5$ MHz. Considering the exact structure geometry, we estimate the effective volume of Brillouin mode to be $V_{eff} = 1.46 \times 10^{-12}$ m^3 along with an effective area $A_{eff} = 155$ μm^2 and an effective length $L_{eff} = 2\pi \times 1.5$ mm. With use of V_{eff} and assuming the overlap factor between pump and Brillouin modes being 0.22, we find the bulk Brillouin center-line gain coefficient to be $g_b \approx 1.32 \times 10^{12}$ Hz/J$^{1/2}$ and the Kerr nonlinearity to be $g_K \approx 2.61 \times 10^{15}$ Hz/J following the formulas given in Ref. 1. Finally, we compute the complex envelope of total intracavity field in Brillouin mode (under a co-rotating frame) with[6],

$$\psi_-(t,\theta) = \sum_n a_{-,n}(t) e^{in\theta}. \tag{S25}$$

Here, θ is the azimuthal angle. Thus, we can calculate the envelope of intracavity energy by $|\psi_-(t,\theta)|^2$.

It is apparent that Fig. 3a, b of the main text perfectly verifies our theory presented in Section I. Since the generated Brillouin laser is also above the threshold of the OPO, after more than 140-μs interaction shown in Fig. 3a, b, the Kerr-comb generation will be then generated and a stable single-soliton state will be subsequently formed around the time $t = 170$ μs. Note that, before the soliton generation, the calculated intracavity energy of the CW background field is as high as ~0.60 nJ (higher than the OPO threshold ~0.56 nJ), however, after the formation of the single soliton, the intracavity energy of the background field is attenuated to as low as ~0.52 nJ (lower

than the OPO threshold), which numerically confirms the strong interaction between the generated soliton microcomb and the background light.

To further demonstrate that the two-soliton state will be forbidden in the Brillouin lasing with the same parameters in Fig. 3a, b of the main text, we numerically feed the system with two solitons and then look at their temporal evolutions (see Fig. 3c of the main text). Here, the initial two-soliton state is a stable state directly simulated (external pump scheme) from the comb modes with the same parameters as the Brillouin mode family in Fig. 3a, b of the main text. In addition, in the course of searching the solution of this two-soliton state, we set the pump power to keep the same amplitude of the CW background field as the soliton state in Fig. 3b of the main text. From Fig. 3c of the main text, one can see that the CW background field decays after a short time (see the red dashed curve in Fig. 3d of the main text). Obviously, two solitons demand more intracavity energy than a single soliton, and propagating one more soliton in the intracavity Brillouin laser will further cause a great reduction in the intracavity energy of the Brillouin wave as evidenced from Eq. (S24). Then, according to Eq. (S22), the decrease of this intracavity energy will result in the reducing in the amplitude of the CW background field. From Fig. 3d of the main text, it can be found that the amplitude of the CW background light is almost zero (outside the soliton locations within the microcavity), which indicates that there is not enough parametric gain available to prevent the damping of the solitons. As illustrated in Fig. 3c of the main text, after few oscillation cycles, the two solitons are eventually diminished into one stable soliton because of the insufficient system energy. To sum up, this two-soliton examination provides another numerical evidence on the monostability feature of the single-soliton state described in Section I.

III. Comparison of the generation of the monostable single soliton to the conventional Kerr solitons

To compare the generation of the single-soliton generation in our system to the conventional Kerr soliton system[7], we attempt to numerically simulate the generation of the single solitons in these two different systems, respectively. In the comparison, the single-soliton formation in the conventional Kerr nonlinear resonators (Fig. S1a) is simulated by the well-known Lugiato–Lefever equations (LLE)[4,8], where the optical modes driven by an external pump laser:

$$\frac{dc_n}{dt} = \left(-i\Delta\omega'_{p,n} - \frac{\gamma'}{2}\right)c_n - i\delta_{0,n}\sqrt{\kappa'_+}s'_{in} - ig'_K\sum_{k,l,m}\delta_{0,n-(k-l+m)}\,c_k c_l^* c_m, \quad \text{(S26)}$$

Here, c_n stands for the amplitude of the comb wave inside the resonator, $\Delta\omega'_{p,n} = \omega'_p - \omega'_n$ with ω'_n denoting the frequencies of the optical resonances ($\omega'_n = \omega'_0 + D'_1 n + \frac{1}{2}D'_2 n^2$, where D'_1 and D'_2 are the FSR and the second-order dispersion), g'_K is the Kerr nonlinear coupling coefficient, and γ' represents the total decay rate of the comb mode.

In Fig. S1b, we simulate a monostable single-soliton state in our system (red line) by using Eqs. (S1)-(S3) with a pump power of 18 mW and pump detuning of $2\pi \times 0.80$ MHz in the Brillouin mode family, as described in Section II. On the other hand, we numerically obtain the same soliton state (blue curve in Fig. S1a) for the conventional soliton microcomb system via Eq. S26. In this simulation, we set the comb modes with the same parameters as the Brillouin modes used in the simulations of Section II, i.e., $\gamma' = 2\pi \times 0.45$ MHz , $\kappa'_+ = 2\pi \times 0.09$ MHz , $D'_2 = 2\pi \times 23.7$ kHz , and $g'_K \approx 2.61 \times 10^{15}$ Hz/J . In addition, we assume the input pump power $|s'_{in}|^2$ to be approximately 7.4 mW and the pump detuning ω'_p to be approximately -2π ×0.62 MHz. At the same time, we plot the corresponding flat state (red line) as a solution of Eq. S26 with the same parameters and under the same pump condition in Fig. S1a. From Fig. S1a, we can see that the intracavity energy of the CW background field for the single soliton state is equal to that of the flat state, and the soliton only locally interacts with the background field in the spatial overlap region. As shown in Fig. S1b, we also calculate intracavity energy for the flat state (red line) before the generation of the singe soliton in our Brillouin-Kerr soliton system, which exhibits a global energy reduction (at every position of the angular coordinate) of the background field after the generation of the single soliton. This behavior is attributed to the strong interaction between the generated Kerr solitons and the CW background light in our Brillouin-Kerr microcomb system.

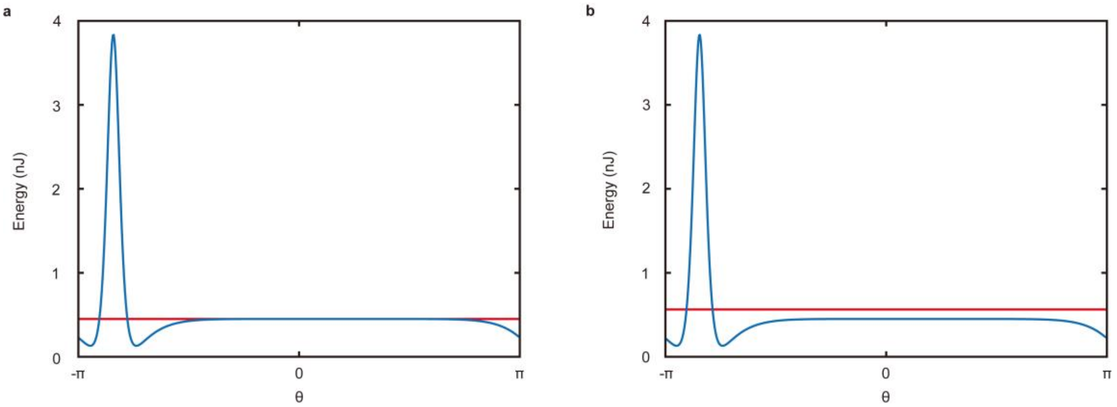

Fig. S1 Numerical simulations on the single-soliton states. a, blue line, conventional single soliton simulated by LLE model; red line, corresponding flat state. **b**, blue line, monostable single soliton excited in Brillouin-Kerr frequency comb system; red line, corresponding flat state before the soliton generation.

To experimentally compare the generation of the monostable single-soliton generation to the conventional Kerr solitons, we directly pump the Brillouin optical mode at 1561.4 nm to generate the conventional Kerr solitons by using an auxiliary laser at 1557.3 nm to suppress the thermo-optic effect[9]. As shown in Fig. S2a, discrete and stochastic soliton steps are obviously observed in the transmission. This phenomenon indicates that the soliton existence range for different states is still in degeneracy. It is worth mentioning that the generated spectra generated here (Fig. S2b) are slightly different from Fig. 2b in the main text due to the increased coupling between the fiber taper and the microcavity.

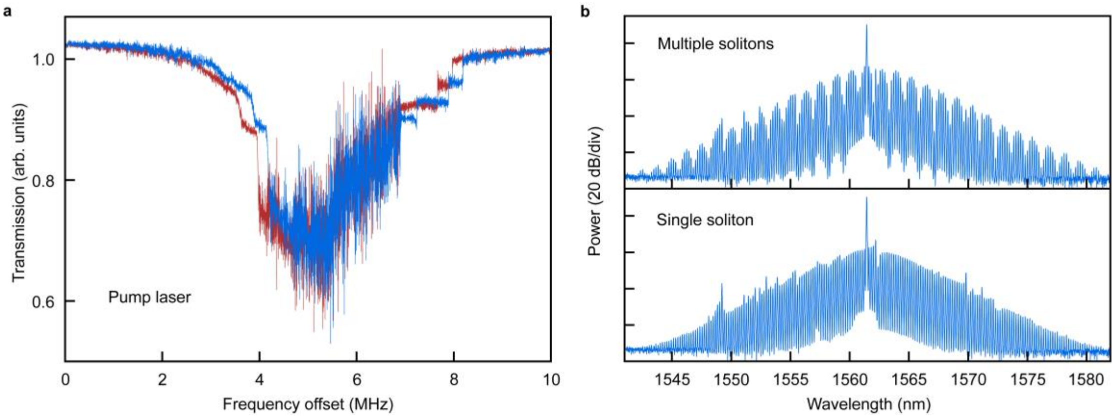

Fig. S2 The generation of conventional Kerr solitons by using an auxiliary laser. a, The measured transmission of pump laser at 1561.4 nm and auxiliary laser at 1557.3nm. The steps existing in the red-detuning regimes of the Brillouin mode. **b**, The measured optical spectra of solitons corresponding to (**a**).

IV. Phase diagram of a Brillouin-Kerr-comb system with monostable single-soliton states

To better understand our system, in this session we have presented the phase diagrams (like Fig. 3e in the main text) by numerically simulating Brillouin-Kerr comb states using the same parameters employed in Section II. Meantime, to have an in-depth understanding of the phases depicted in Fig. 2 of the main text, we have particularly calculated the Brillouin laser and Brillouin-Kerr comb states under the 18.0-mW pump power. As illustrated in Fig. S3, by gradually decreasing the input pump frequency, we can observe that the intracavity energy of the Brillouin laser states increases monotonically, transits from the region of the flat states (Phase IV) into the region of the single-soliton and flat states (Phase V), and then passes the OPO threshold. Above the OPO threshold, along with the increase of the pump frequency, the system sequentially goes through the monostable single-soliton state (Phase I), the hopping state in between single- and two-soliton states (Phase II), and the two-soliton breather state (Phase III), and then enters the region (Phase VI) of chaotic oscillations of the Brillouin laser and Kerr combs. These distinct system states agree very well with our experimental measurements (see Fig. 2 in the main text).

Furthermore, as demonstrated in Fig. S4, we have additionally shown an example of the calculated transmission powers by scanning pump laser frequency detuning at the 18.0-mW pump power. From Fig. S4, one can see that the system will start from the region with no Brillouin lasing into the Brillouin laser, and then sequentially pass the Phases I-III. Note that the soliton states in the region V of Fig. S3 will not be excited in the operation for the bi-stable property of the region. Interestingly, these phase transitions in our calculations match well with our experimental results, as shown in Fig. 2a of the main text. Here, we would like to point out that the deviations between Fig. S4 and Fig. 2a of the main text mainly stem from the neglecting of thermo-optic effect[10] in the simulation.

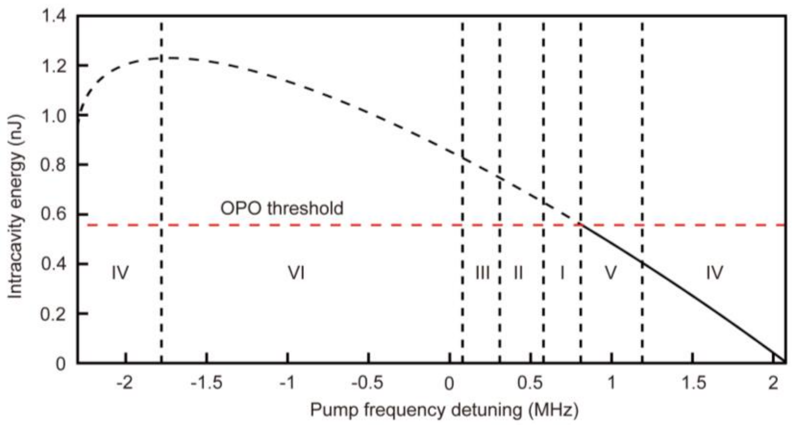

Fig. S3 The intracavity energy of the Brillouin wave as a function of the input pump frequency detuning. The black solid (dashed) curve delineates the steady state below (above) the threshold of the optical parametric oscillation (OPO). Capital Roman numerals label different pump frequency detuning regions, whose phases correspond to the experimentally observed phases in Fig. 2 of the main text: (I) monostable single-soliton state; (II) hopping state between the single- and two-solitons; (III) breathing two-soliton state. The other phase domains labelled by Roman numerals are, respectively, (IV) flat states; (V) coexistence of the single-soliton and flat states; (VI) chaotic states.

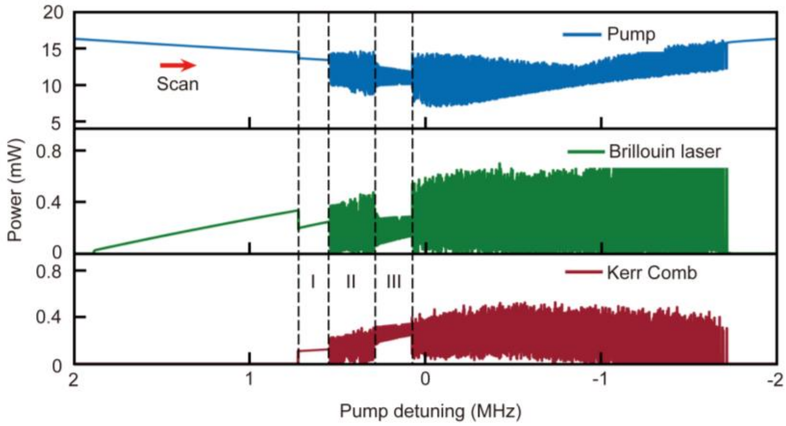

Fig. S4 Simulated transmitted powers. The input pump (blue), generated Brillouin laser (green), and generated combs (red) by scanning the pump frequency from detuning 2 to -2 MHz at the pace of 400 MHz per second. The regions labeled with I-III indicate the corresponding state regions in Fig. S3.

To have an intuitive picture of the states in different phase regions, in Fig. S5a, b, we have reported some theoretical results for the Brillouin-Kerr comb states in Phase II of Fig. S3 (or Phase II in Fig. 3e of the main text), which clearly exhibit that the system hops between single and two solitons. In Fig. S5c, d, we have also displayed the typical numerical simulations on the breathing two-solitons (corresponding to Phases III in Fig. S3 or Fig. 3e of the main text). The calculated temporal traces of the soliton hopping (Fig. S5b) and breathing (Fig. S5d) agree well with the experimental measurements shown in Fig. 2c of the main text. Intriguingly, one may find that the chaotic states (corresponding to Phase VI in Fig. S3) shown in Fig. S5e, f behave different from the typical modulation instability states or spatio-temporal chaos[11], because of observing of chaotic temporal oscillations of the background field and

stochastic switching of soliton numbers (see Fig. S5e). This observation is related to the interaction between the solitons and the background field in our Brillouin-Kerr soliton system. The detailed study on such chaotic motions is beyond the scope of this work, which will be further studied in the future. In the experiment, we did not observe the soliton crystals (or Turing rolls), which may be attributed to the limited frequency range (or the red detuning) of the generated Brillouin laser. This is because the frequency tuning range of the generated Brillouin laser is much smaller than the input pump[1].

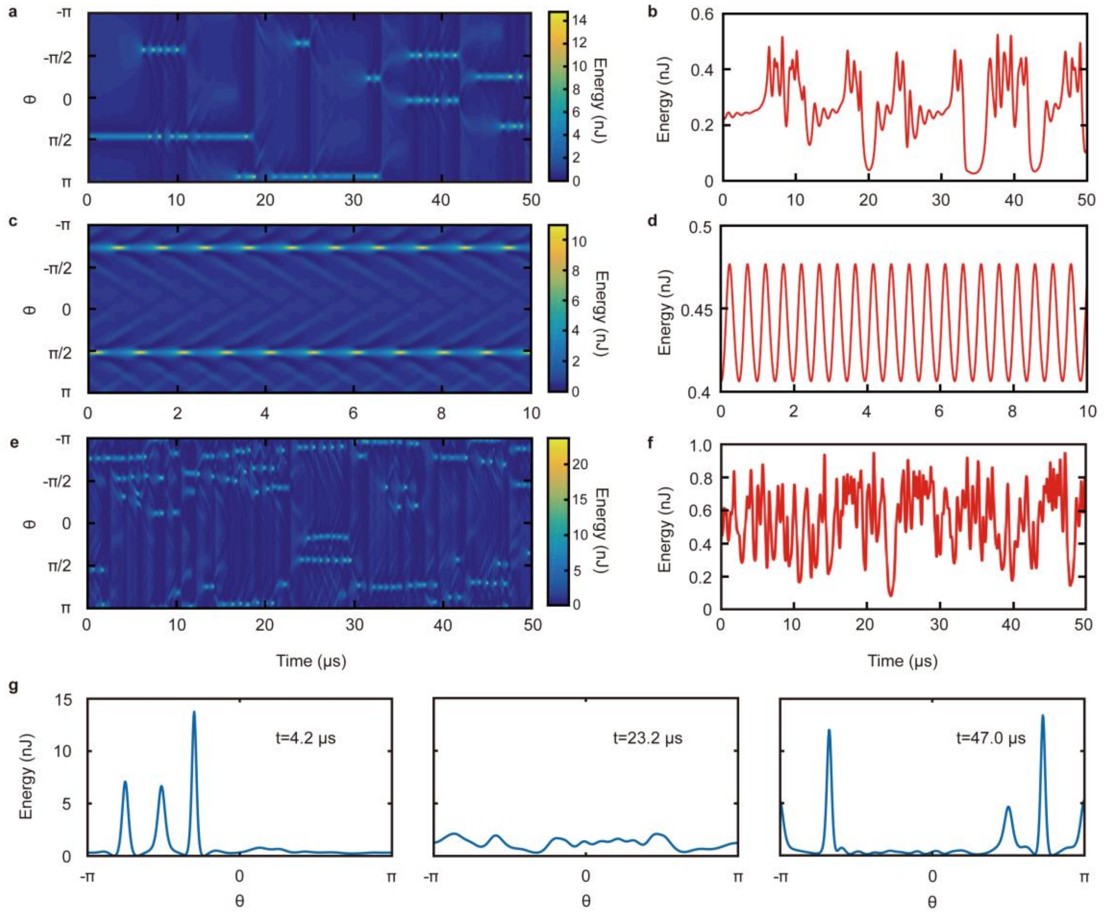

Fig. S5 Numerical simulations of the states in different phase regions. a, **b**, Representative numerical simulations on a typical hopping state between single- and two-soliton states, $\Delta\omega_p = 2\pi \times 0.40$ MHz. **c**, **d**, Representative numerical simulations on breathing two solitons, $\Delta\omega_p = 2\pi \times 0.30$ MHz. **e**, **f**, Representative numerical simulations on chaotic Brillouin-Kerr combs, $\Delta\omega_p = 2\pi \times -0.50$ MHz. **a**, **c**, **e**, Temporal evolutions of intracavity-energy envelope of total optical field in Brillouin mode. **b**, **d**, **f**, Temporal evolutions of intracavity energies of total comb waves. **g**, Intracavity-energy envelope of total optical field in Brillouin mode at different time in (**e**).

V. The emergence of the monostable single soliton determined by Q-factor

To further explain the strong interaction between the generated soliton and the background field, we have numerically calculated the emergence of the monostable single soliton as a function of the loaded Q-factor of the Brillouin optical mode, under

the condition of unchanged system parameters except for the intrinsic decay rate of the Brillouin mode γ_{i-}. Here, we compute this Q-factor with $Q_L = \frac{\omega_{-,0}}{\gamma_-}$. It shows that the monostable single soliton will appear given that the loaded Q-factor is larger than 3.3×10^8. This behavior can be attributed to the fact that the higher Q-factor of the Brillouin optical mode will lead to a stronger interaction between the generated soliton and the CW background field (see Eq. (24) and its associated discussions). As a result, above a certain Q_L, the strong interaction between the generated soliton and the background field induces the emergence of the monostable single-soliton states.

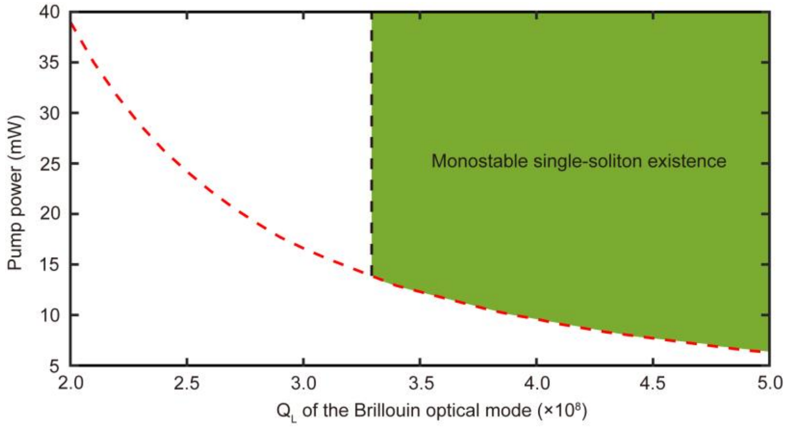

Fig. S6 The generation of the monostable single soliton determined by Q-factor. The existence diagram of the monostable single-soliton states as a function of input pump power and the loaded Q-factor of the Brillouin optical mode. Dashed red curve plots the OPO threshold of the Brillouin laser under the optimal pump detuning.

VI. Reliability on generation of single-soliton states

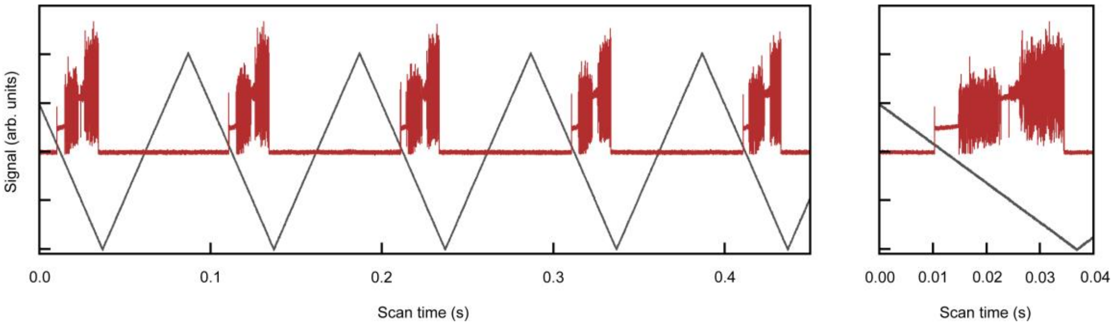

Fig. S7 The deterministic generation of the single-soliton states. (Left) The comb power trace (red curve) displays repeated single-soliton generation. (Right) A zoomed-in view of partially selected signal. The gray curves indicate the triangle scan voltage.

In the experiment, we have repeatedly swept the pump frequency 100 times with a triangle signal to verify the deterministic generation of the single-soliton states, and have 100% successfully generated the steps of the single-soliton states. In Fig. S7, as an example, we have shown five consecutive times of the single-soliton generations from the measured 100-time successful generations. From the data, one may find that the generations of single soliton are very reliable.

VII. Evolution of Brillouin laser power against input pump power

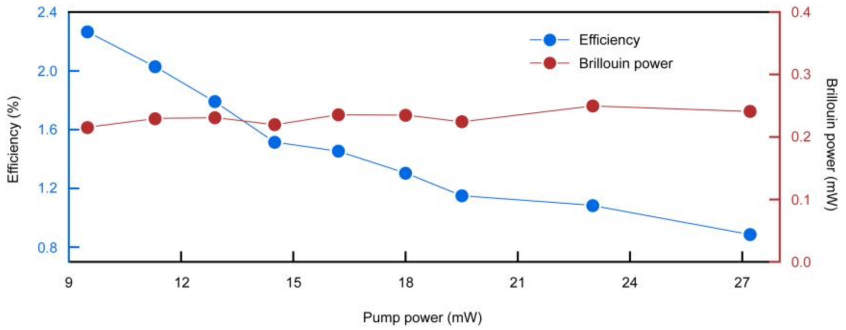

Fig. S8 The output Brillouin laser power after single soliton generation. Power (red) and efficiency (blue) curves of Brillouin laser versus the pump power.

We achieve monostable single solitons within a wide range of input pump power under fixed coupling conditions of the cavity modes. At the same time, we record the output Brillouin laser power after single soliton generation. As shown in Fig. S8, our experimental data show that the output power of the Brillouin laser is nearly unchanged when increasing the input pump power, which indicates a power clamping of the Brillouin laser during the single-soliton generation.

VIII. Close-up spectra of generated single-soliton microcomb

In Fig. S9 illustrates the measured forward optical spectrum and zoom-in backward optical spectrum. From Fig. S9, we can deduce that the single-soliton state is generated via the first-order Brillouin laser and there is no cascaded Brillouin laser generated. Also, in the forward direction, some portions of the reflected comb lines are observable due to the Rayleigh backscattering of the microtoroid resonator.

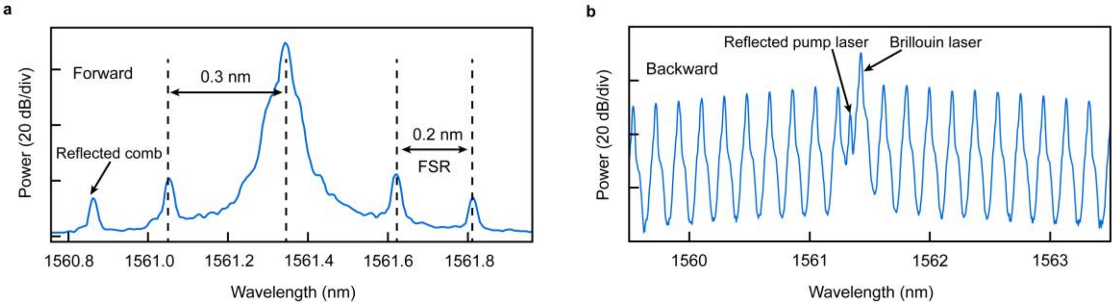

Fig. S9 The measured forward optical spectrum and zoom-in backward optical spectrum. Forward optical spectrum (**a**) and zoom-in backward optical spectrum (**b**) with the single soliton state.

IX. Fundamental linewidth measurement of the Brillouin laser

Figure S10 shows the measured fundamental linewidths of Brillouin laser with different input pump power after the single solitons are generated. Here, the measured linewidths of the Brillouin laser are slightly narrower than those obtained from the turnkey single-soliton microcomb (Fig. 5 of the main text), which is attributed to the

different coupling conditions of the tapered fiber. Actually, the coupling condition is the same to the measurements of Figs. 2-4 in the main text. Also, for the generation of the low-noise microwave signal, the tapered fiber is touched to the microresonator to enhance the system stability.

In previous Brillouin lasers studies[12], the fundamental linewidth of the Brillouin laser is often inversely proportional to the input pump power. This does not apply to our case. As shown in Fig. S10, our measurement unveils that the fundamental linewidth of the Brillouin laser in our work in nearly independent of the pump power. This is because the generated Brillouin laser is clamped by the single-soliton generations (see Fig. S8).

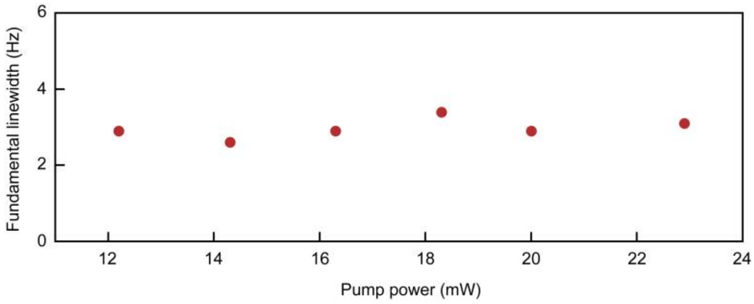

Fig. S10 The measurement of the fundamental linewidth. Fundamental linewidths of Brillouin laser versus the pump power.

X. Dispersion measurement of pump and Brillouin modes

Figure S11 shows the measured the frequency dispersion curves of the Brillouin and pump mode families. As shown in Fig. S11b, the dispersion of the Brillouin mode family is anomalous with the second-order dispersion parameter $D_2/2\pi$ of ~23.7 kHz. In contrast, the frequency dispersion curve of the pump mode family is strongly distorted by the avoided mode crossing (see Fig. S11a). In our experiment, we did not observe Kerr comb generation in the mode family of the pump mode due to the strongly distorted dispersion and the relatively low Q-factor of the pump mode.

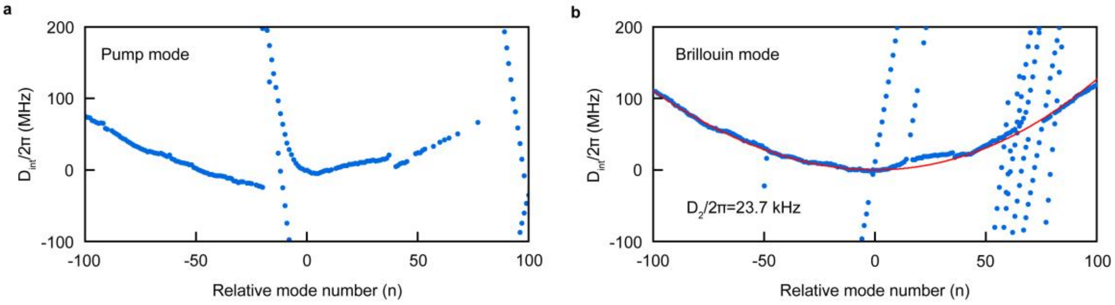

Fig. S11 The measurement of dispersion. Measured frequency dispersions for the mode families of pump (**a**) and Brillouin (**b**) modes.

XI. The measurements under high pump power

In experiment, we also explore the generation of the Brillouin-Kerr solitons by increasing the input pump power up to ~100 mW. The experimental results are presented in Fig. S12. As one can see the monostable single soliton still persists even under such high pump power. Note that this is different from our previous work[1] where the multi-soliton states were initially emerged with the decreasing the pump frequency.

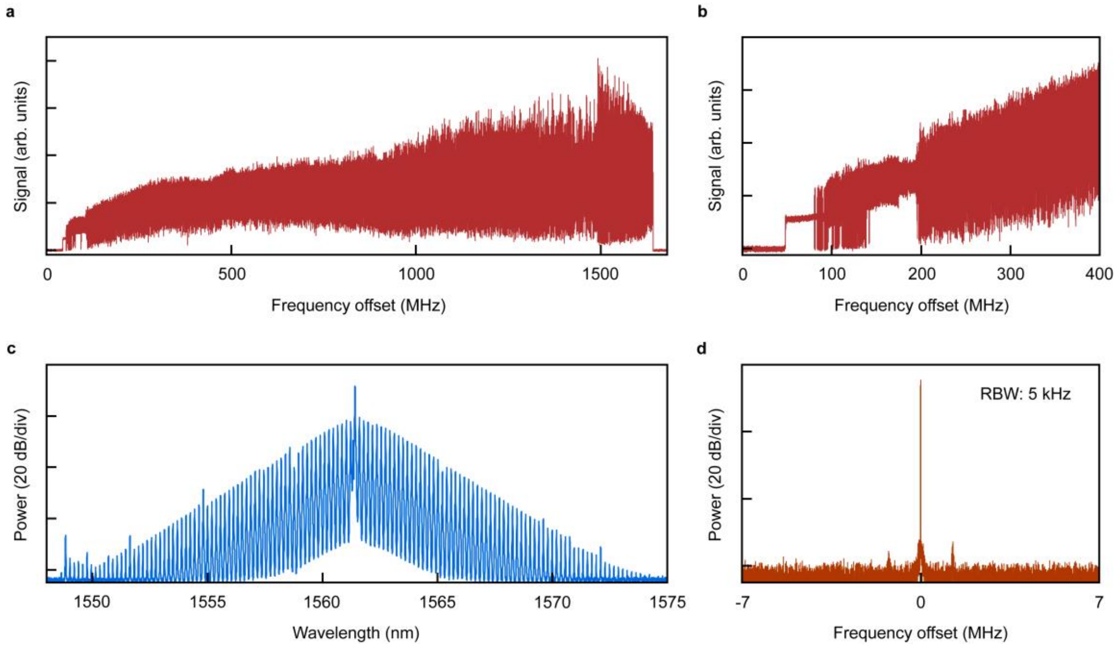

Fig. S12 The generation of the Brillouin-Kerr solitons under high input pump power. a, Typical transmission power spectrum of the generated comb during the scanning process under the pump power of ~100 mW. **b**, The Zoom-in spectrum of the transmission power spectrum shown in (**a**). **c**, Optical spectrum of the monostable single soliton. **d**, Corresponding radiofrequency spectrum of the monostable single soliton with a 5-kHz resolution bandwidth (RBW).

XII. Detuning measurement of the Brillouin laser

To measure the detuning of the generated Brillouin laser during the generation of the monostable single soliton, we employ a weak probe laser to scan the Brillouin cavity mode[13] (see Fig. S13a). The back-reflected Brillouin laser produces a beat note with the probe laser. Tracking the disappearance position of the beat note provides insight into the Brillouin laser detuning. As the pump laser and probe laser (~10.7 GHz shifts from the pump frequency) propagate in the same direction, we further filter out the pump laser through a fiber Bragg grating (0.2-nm bandwidth). This approach confirms that the Brillouin laser is red-detuned (the resonator frequency higher than Brillouin laser frequency, see Fig. S13b) during soliton microcomb generation.

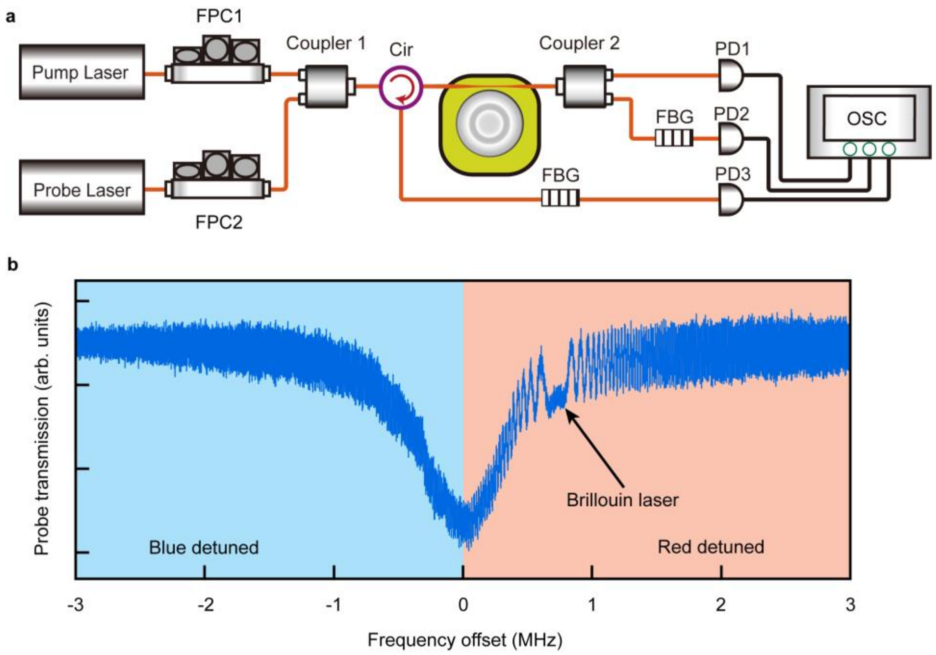

Fig. S13 The measurement of the generated Brillouin laser detuning during the generation of the monostable single soliton. a, Experimental set-up for Brillouin laser detuning measurements. FPC, fiber polarization controller; Cir, circulator; FBG, fiber Bragg grating; PD, photodiode; OSC, oscilloscope. **b**, Typical transmission spectrum obtained by sweeping the Brillouin mode with a probe laser after generating a single soliton.

XIII. Comparison table of phase-noise performance from microcombs

We compare our low-noise, turnkey microwave source based on the Brillouin-Kerr soliton microcomb with other microcomb-based microwave oscillators[1,14-22], which are scaled to the same carrier frequency of 23.3 GHz. Several material platforms such as silica, silicon nitrides, and bulk magnesium fluoride (MgF_2) crystal are included. The results are tabulated in Table S1 below.

Table S1 Comparison of phase-noise

Material	Configuration	Carrier freq. (GHz)	Turnkey operation	SSB phase noise (dBc/Hz, scaled to 23.3 GHz) 1 kHz	10 kHz	Reference
SiO_2	Bright soliton	23.3	W/	-94	-128	This work
SiO_2	Bright soliton	10.4	W/o	-81	-123	1
SiO_2	Bright soliton	22	W/o	-109	-124	14
SiO_2	Bright soliton	11.4	W/o	-101	-124	15
SiO_2	Bright soliton	15.2	W/o	-86	-110	16
SiO_2	Bright soliton	10	W/o	-93	-118	17
Si_3N_4	Bright soliton	19.6	W/o	-79	-109	18
Si_3N_4	Dark soliton	5.4	W/	-71	-101	19
Si_3N_4	Bright soliton	560	W/o	-105	-127	20
MgF_2	Bright soliton	9.9	W/o	-107	-120	21
MgF_2	Bright soliton	14	W/o	-107	-131	22

References

1. Bai, Y. et al. Brillouin-Kerr soliton frequency combs in an optical microresonator. *Phys. Rev. Lett.* **126**, 063901 (2021).
2. Haus, H. A. Waves and Fields in Optoelectronics (Prentice-Hall, Englewood, 1984).
3. Wabnitz, S. Suppression of interactions in a phase-locked soliton optical memory. *Opt. Lett.* **18**, 601-603 (1993).
4. Parra-Rivas, P., Gomila, D., Matías, M. A., Coen, S. & Gelens, L. Dynamics of localized and patterned structures in the Lugiato-Lefever equation determine the stability and shape of optical frequency combs. *Phys. Rev. A* **89**, 043813 (2014).
5. Hansson, T., Modotto, D. & Wabnitz, S. On the numerical simulation of Kerr frequency combs using coupled mode equations. *Opt. Commun.* **312**, 134-136 (2014).
6. Godey, C., Balakireva, I. V., Coillet, A. & Chembo, Y. K. Stability analysis of the spatiotemporal Lugiato-Lefever model for Kerr optical frequency combs in the anomalous and normal dispersion regimes. *Phys. Rev. A* **89**, 063814 (2014).
7. Kippenberg, T. J., Gaeta, A. L., Lipson, M. & Gorodetsky, M. L. Dissipative Kerr solitons in optical microresonator. *Science* **361**, eaan8083 (2018).
8. Herr, T. et al. Temporal solitons in optical microresonators. *Nat. Photon.* **8**, 145-152 (2014).
9. Zhang, S. et al. Sub-milliwatt-level microresonator solitons with extended access range using an auxiliary laser. *Optica* **6**, 206 (2019).
10. Carmon, T., Yang, L. & Vahala, K. J. Dynamical thermal behavior and thermal self-stability of microcavities. *Opt. Express* **12**, 4742 (2004).
11. Coulibaly, S. et al. Turbulence-induced rogue waves in Kerr resonators. *Phys. Rev. X* **9**, 011054 (2019).
12. Li, J. et al. Characterization of a high coherence, Brillouin microcavity laser on silicon. *Opt. Express* **20**, 20170-20180 (2012).
13. Del'Haye, P. et al. Phase steps and resonator detuning measurements in microresonator frequency comb. *Nat. Commun.* **6**, 5668 (2015).
14. Kwon, D. et al. Ultrastable microwave and soliton-pulse generation from fibre-photonic-stabilized microcombs. *Nat. Commun.* **13**, 381 (2022).
15. Yao, L. et al. Soliton microwave oscillators using oversized billion Q optical microresonators. *Optica* **5**, 561-564 (2022).
16. Yang, Q.-F. et al. Dispersive-wave induced noise limits in miniature soliton microwave sources. *Nat. Commun.* **12**, 1442 (2021).
17. Nie, M. et al. Synthesized spatiotemporal mode-locking and photonic flywheel in multimode mesoresonators. *Nat. Commun.* **13**, 6395 (2022).
18. Liu, J. et al. Photonic microwave generation in the X- and K-band using integrated soliton microcombs. *Nat. Photon.* **14**, 486-491 (2019).
19. Jin, W. et al. Hertz-linewidth semiconductor lasers using CMOS-ready ultra-high-Q microresonators. *Nat. Photon.* **15**, 346-353 (2021).
20. Kuse, N. et al. Low phase noise THz generation from a fiber-referenced Kerr microresonator soliton comb. *Commun. Phys.* **5**, 312 (2022).
21. Liang, W. et al. High spectral purity Kerr frequency comb radio frequency photonic

oscillator. *Nat. Commun.* **6**, 7957 (2015).

22. Lucas, E. et al. Ultralow-noise photonic microwave synthesis using a soliton microcomb-based transfer oscillator. *Nat. Commun.* **11**, 374 (2020).
